# Adalimumab-Induced Thrombocytopenia in a Patient With Hidradenitis Suppurativa

**DOI:** 10.7759/cureus.14769

**Published:** 2021-04-30

**Authors:** Amna Al-Tkrit, Zaid Obada, Sara Muqeet, Jose Cervantes

**Affiliations:** 1 Internal Medicine, Jamaica Hospital Medical Center, Queens, USA; 2 Hematology and Medical Oncology, Jamaica Hospital Medical Center, Queens, USA

**Keywords:** adalimumab, anti-tumor necrosis factor-alpha (anti-tnf-alpha), thrombocytopenia, platelet count, hematological abnormalities, drug-induced thrombocytopenia (dit), hidradenitis suppurativa, intravenous immunoglobulin (ivig)

## Abstract

Adalimumab-induced thrombocytopenia is a rarely occurring condition that may present with hemorrhagic manifestations. This report describes a case of a patient who presented with severe, symptomatic thrombocytopenia while on adalimumab for the treatment of hidradenitis suppurativa. The patient responded to treatment with steroids, intravenous immunoglobulin (IVIG), and platelet transfusion, in addition to discontinuation of adalimumab.

## Introduction

Thrombocytopenia is a rare side effect associated with the use of anti-tumor necrosis factor-alpha (anti-TNF-alpha) agents such as adalimumab. Patients may be asymptomatic or present with mild-to-severe bleeding. Only a few case reports of adalimumab-induced thrombocytopenia in patients using the medication for various inflammatory conditions, such as Crohn’s disease and psoriasis, can be found in the literature [1]. In this report, we present the case of a young patient with a two-year history of adalimumab use for the treatment of hidradenitis suppurativa, who presented with diffuse petechial hemorrhages, mild gingival bleeding, and a severely low platelet count. A diagnosis of adalimumab-induced thrombocytopenia was made after excluding all other potential causes of thrombocytopenia.

## Case presentation

A 29-year-old man with a past medical history of hidradenitis suppurativa, for which he was taking adalimumab, presented to the emergency department with the complaint of having red spots on his arms and legs. The spots appeared suddenly five days ago. He first noticed raised, small, red-colored spots on his buccal mucosa and then found small, diffuse, red spots all over his body. The patient also reported that he had observed mild bleeding while brushing his teeth. He denied bleeding from any other body sites. The patient had not experienced similar symptoms before, and there was no history of joint pain or joint swelling, liver disease, or HIV infection. The patient was diagnosed with hidradenitis suppurativa five years ago and had been taking adalimumab daily for the last two years. He never had any complications associated with the use of this medication and denied any recent changes in its dosage. The patient also denied starting any new medications in the recent past. His family history was unremarkable for any bleeding disorder.

On examination, the patient was in no acute distress. Vitals were as follows: temperature of 99.7°F, blood pressure of 144/87 mm Hg, pulse rate of 89 beats/min, and respiratory rate of 20 breaths/min. Diffuse petechiae were noted to be present over the skin. No lymphadenopathy or hepatosplenomegaly was present. The initial labs revealed a platelet count of 1,000/mm^3^. Other lab values included hemoglobin of 13.9 g/dL, hematocrit 39.8%, white blood cell count of 12,600/mm^3^, reticulocyte count 1%, international normalized ratio (INR) of 1.2, and partial thromboplastin time (PTT) of 30 seconds. Liver function tests were normal. The urinalysis was positive for red blood cells. The peripheral blood smear confirmed thrombocytopenia with no platelet clumping, schistocytes, or any other abnormalities. The blood cultures and urine cultures did not show any growth. An abdominal ultrasound was performed, which was found to be unremarkable for any intra-abdominal abnormalities.

After ruling out all the other causes of thrombocytopenia, a diagnosis of exclusion of adalimumab-induced thrombocytopenia was made. The medication was discontinued and the patient was given intravenous immunoglobulin (IVIG) 1 g/kg/day for two days along with oral dexamethasone 40 mg daily for four days. One unit of platelets was also transfused. The platelet count responded appropriately to the treatment and was found to be 74,000/mm^3^ on the day of discharge from the hospital four days later. The patient was discharged in a medically stable condition and was advised to follow up with a hematologist to monitor complete resolution of thrombocytopenia and with his rheumatologist to consider replacing adalimumab with an alternative medication.

## Discussion

Drug-induced thrombocytopenia (DIT) is a serious adverse reaction that may be associated with the use of several different medications. It has been estimated that approximately 25% of critically ill patients are at an increased risk of developing DIT and that the overall worldwide incidence can be as high as 10 cases per million people per year. The condition typically presents as an acute and severe reduction in platelet count, with values usually below 50,000/mm^3^; thus, potentially increasing the risk of spontaneous, life-threatening bleeding [2]. DIT may occur through a variety of underlying mechanisms, such as an impairment in platelet production due to bone marrow suppression, or an accelerated clearance of platelets from the circulation due to nonimmune, immune-mediated, or autoimmune mechanisms [2-4].

Anti-TNF-alpha agents, such as etanercept, infliximab, and adalimumab, are being increasingly used for the management of inflammatory, immune-mediated diseases. Adalimumab is a human recombinant immunoglobulin G (IgG) monoclonal antibody that binds to TNF-alpha with high affinity. The drug acts by inhibiting the binding of TNF-alpha to p55 and p75 receptors located on the surface of target cells. Like the other anti-TNF-alpha agents, adalimumab is used for the treatment of inflammatory conditions such as rheumatoid arthritis, ankylosing spondylitis, and psoriatic arthritis [5,6]. In addition, adalimumab is the only drug approved by the FDA for the treatment of moderate-to-severe hidradenitis suppurativa, a chronic, relapsing, inflammatory disorder characterized by the presence of painful nodules, abscesses, sinus tracks, and scarring mainly in the inguinal and axillary regions [7,8]. The safety and limited hematological toxicity of anti-TNF-alpha agents are well established; however, these drugs are known to block stem cell differentiation and to affect bone marrow signaling and thus can potentially result in numerous hematological complications. Indeed, several non-malignant, hematological adverse events, some with fatal outcomes, have been reported in patients receiving these medications. These include thrombocytopenia, neutropenia, aplastic anemia, pancytopenia, and hypercoagulability [1].

Thrombocytopenia, as defined by a platelet count of less than 150,000/mm^3^, is an extremely rare adverse event associated with the use of anti-TNF-alpha agents. Severe thrombocytopenia (platelet count less than 50,000/mm^3^) is even rarer, and most of the literature is limited to case reports [1,9]. In one retrospective study, no patients who received anti-TNF-alpha agents had a lowering of the platelet count below 50,000/mm^3^ or displayed any clinical manifestations of thrombocytopenia [10]. However, in another study, 5.97% of the patients were reported to have a platelet count below 50,000/mm^3^ and three patients were found to be symptomatic [11]. In comparison to infliximab, adalimumab appears to be less commonly associated with thrombocytopenia. Data collected by the drug manufacturer during clinical trials showed that the incidence of thrombocytopenia (platelet count < 100,000/mm^3^) was approximately 0.1% with adalimumab, whereas the incidence was between 0.5% and 1.9% in the case of infliximab [1].

The exact mechanism by which adalimumab and other anti-TNF-alpha agents cause thrombocytopenia is unclear, and several possible mechanisms have been put forward. For instance, it has been proposed that these medications may be associated with the production of anti-platelet antibodies that bind to glycoproteins located on the cell membrane of platelets and result in accelerated destruction of platelets. However, the presence of these antibodies has never been firmly demonstrated [9]. According to another hypothesis, anti-TNF-alpha drugs may induce the formation of immune complexes that bind to the platelet surface, thus activating the complement system and causing subsequent platelet destruction. Another hypothesis involves the induction of Th1 lymphocyte apoptosis by anti-TNF-alpha drugs. This may result in a relative excess of Th2 lymphocytes, which, in turn, stimulate the production of antiplatelet antibodies, thus causing platelet destruction and thrombocytopenia [12,13]. Moreover, since TNF-alpha is involved in the regulation of some pro-inflammatory cytokines, such as granulocyte-macrophage-colony-stimulating factor (GM-CSF) and interleukins (ILs) including IL-1, IL-6, and IL-8, the anti-TNF-alpha agents can potentially block stem cell differentiation and thus lead to bone marrow failure [12,14]. According to some studies, thrombocytopenia induced by anti-TNF-alpha agents may be an idiosyncratic reaction occurring in genetically predisposed individuals, whereas others believe that some unknown autoimmune mechanisms associated with the production of autoantibodies during apoptosis may contribute to the decrease in the platelet count [13]. Other possible mechanisms include direct marrow toxicity or a lupus-like syndrome [14]. However, it is likely that more than one mechanisms contribute to the development of thrombocytopenia in patients receiving anti-TNF-alpha agents [13].

Thrombocytopenia can present with a wide range of clinical manifestations. Patients may be asymptomatic or present with petechial hemorrhages, nonpalpable purpura, ecchymosis, oozing of blood from the buccal mucosa, or severe bleeding. Most severe symptoms typically occur when the platelet count falls below 20,000/mm^3^ [15]. Thrombocytopenia caused by anti-TNF-alpha agents has a variable time of onset and has been reported to occur from less than one day to three years after starting the use of these medications. Clinical improvement is often seen after the medication is stopped, and reexposure to the drug is often associated with a relapse [16]. The complete blood count (CBC) and peripheral blood smear analysis are important in the evaluation of DIT. These are rapid and inexpensive tests, which can help rule out pseudothrombocytopenia caused by in vitro platelet aggregation and can also identify if the thrombocytopenia is isolated or is associated with a suppression of the other cell lines. The diagnosis of DIT can be confirmed by demonstrating the presence of platelet-bound or platelet-specific antibodies produced by exposure to a certain drug. Flow cytometry, enzyme immunoassays (EIAs), glycoprotein (GP)-specific assays, platelet suspension immunofluorescence test, and antiglobulin-based assays are some of the methods that are used for the detection of drug-dependent antibodies. However, false-negative results may be seen due to the low sensitivity of some of these diagnostic methods for some antibodies [2]. Moreover, these tests may not be readily available in all instances [15].

The management of DIT is based on its severity and the occurrence of symptoms. Discontinuation of the causative agent along with clinical and laboratory follow-up may be the only actions needed to manage patients with mild-to-moderate thrombocytopenia (platelet count more than 50,000/mm^3^). However, specific treatment in addition to discontinuation of the offending drug may be required if the platelet count is below 50,000/mm^3^, particularly when it is associated with clinical manifestations. The pharmacological management of adalimumab-induced thrombocytopenia is similar to that of immune thrombocytopenic purpura, as an immune mechanism is thought to be involved in the pathogenesis of both these conditions. The first-line treatment consists of administering corticosteroids [12]. Platelet transfusion is not typically indicated in patients with DIT, as there is continued immune-mediated destruction of the platelets in the presence of the causative drug. However, transfusion of platelets can be effective after discontinuation of the offending agent and may be considered if the platelet count is below 20,000/mm^3^ and severe bleeding is present [15]. Patients who require rapid platelet increment or do not respond to corticosteroid therapy may be treated with IVIG. The use of rituximab and mycophenolate mofetil has also been reported [12].

Anti-TNF-alpha agents, such as adalimumab, are considered to be a rare cause of thrombocytopenia. The literature contains only a limited number of case reports of adalimumab-induced thrombocytopenia in patients who were using the medication for the treatment of Crohn’s disease and psoriasis [1]. To our knowledge, this is the first case of adalimumab-induced thrombocytopenia in a patient who was taking adalimumab for the treatment of hidradenitis suppurativa. The patient presented with diffuse petechial hemorrhages and mild gingival bleeding and had severe thrombocytopenia, as evidenced by a platelet count of 1,000/mm^3^. Microscopic hematuria was also noted to be present. The probable causal relationship between adalimumab and thrombocytopenia was supported by the fact that the patient had been taking adalimumab for two years and was not taking any other medications that could have been associated with the development of thrombocytopenia. Moreover, sustained platelet recovery was seen after the discontinuation of adalimumab. All other possible causes, including pseudothrombocytopenia, were also excluded. Adalimumab was discontinued and the patient was started on IVIG and oral steroids. Platelet transfusion was also performed. The patient responded to these treatment interventions, and a significant improvement in the patient’s platelet count was observed within a period of four days.

This case report highlights the fact that while the safety of anti-TNF-alpha agents is well established, the precise interaction of these medications with the hematopoietic and immune systems is not completely understood. This may make it difficult to predict the adverse effects associated with these agents. Isolated thrombocytopenia caused by anti-TNF-alpha therapy is extremely rare; nevertheless, it may be advisable to obtain a baseline platelet count before and shortly after the initiation of these drugs, and regular monitoring of the platelet counts may be taken into consideration, to prevent the occurrence of any serious adverse events. If thrombocytopenia is noted to be present during the course of treatment with anti-TNF-alpha agents, a hematologist should be involved and a thorough investigation for secondary etiologies, such as autoimmune disorders and viral diseases, should be carried out. Additional studies to allow a better understanding of the interaction between anti-TNF-alpha agents and the immune system are warranted.

## Conclusions

Adalimumab is an anti-TNF-alpha agent, which is rarely associated with the development of hematological abnormalities such as thrombocytopenia. Adalimumab-induced thrombocytopenia may sometimes present with hemorrhagic manifestations, and, thus, establishing an early diagnosis is critical. This may be challenging as the condition has a variable time of onset and may develop up to three years after the initiation of the anti-TNF-alpha drug. Therefore, a high index of suspicion should be maintained and a comprehensive workup should be conducted to exclude all other possible underlying etiologies. Platelet recovery is typically seen after the discontinuation of the medication; however, steroids, IVIG, and platelet transfusion may be required in severe cases.
